# LPS ligand and culture additives improve production of monomeric MD-1 and 2 in *Pichia pastoris* by decreasing aggregation and intermolecular disulfide bonding

**DOI:** 10.1016/j.pep.2010.11.018

**Published:** 2011-04

**Authors:** Kristen E. Mengwasser, Clare E. Bryant, Nick J. Gay, Monique Gangloff

**Affiliations:** a Department of Biochemistry, University of Cambridge, 80 Tennis Court Road, Cambridge CB2 1GA, United Kingdom; b Department of Veterinary Medicine, University of Cambridge, Madingley Road, Cambridge CB3 0ES, United Kingdom

**Keywords:** MD-1, MD-2, Aggregation, Disulfide bonds, *Pichia pastoris* expression, Additive screening, LPS binding

## Abstract

Myeloid differentiation proteins MD-1 and MD-2 have both been shown to form a heterogeneous collection of oligomers when expressed in absence of their respective receptor, RP105 and TLR4. The biological relevance of these oligomers is not clear. Only monomeric proteins have been found to be active and able to trigger an immune response to endotoxin by modulating the TLR4 pathway. In this study, we produced variants of MD-1 and MD-2 in *Pichia pastoris*. To minimize the time and expense of initial expression tests, small-scale cultures have been set up to allow the rapid identification of the highest expressing clone and the optimal expression conditions. The expression vectors used, the site of linearization and the locus of integration affected the yield of transformation. Next we screened culture additives and found that they significantly increased the fraction of monomeric proteins secreted in the culture medium (up to 15% of the total MD protein produced). We confirmed their presence by size-exclusion chromatography. Optimal anti-aggregation agents were protein-dependent except for LPS that presented stabilizing effects for all MD proteins. Contrary to previous reports, this study suggests that MD-1 can bind to LPS.

## Introduction

Myeloid differentiation proteins MD-1 and MD-2 are disulfide-rich glycoproteins of 20–25 kDa, tethered to the cell surface by type 1 immune receptors radioprotective protein RP105 and Toll-like receptor TLR4, respectively [1]. Lipopolysaccharide (LPS)^1^ shed by Gram-negative bacteria is recognized by MD-2, which contains the main if not the only binding site amongst all four proteins. LPS binding to MD-2 triggers an immune response by changing the quaternary structure of the TLR4–MD-2 receptor complex [2,3]. Signal transduction is then mediated by TLR4 and ultimately activates over a thousand genes to fight the microbial infection [4,5]. MD-1 is a distant homologue of MD-2 (18% identity) and RP105 has structural similarities to TLR4. Although RP105 conserves the extracellular leucine-rich repeats (LRRs), to which MD-1 binds, it lacks the intracellular signaling domain (the Toll-Interleukin-1 Receptor TIR domain) and contains instead a short cytoplasmic tail. The physiological roles of MD-1 and RP105 are not clear. A host of controversial findings suggest either positive or negative regulation capacities of MD-1 and RP105 on TLR4 signalling [6,7]. In a recent study, evidence has been presented that MD-1-RP105 cannot bind LPS in contrast to MD-2-TLR4 and that these proteins are sequestered in an inactive tetrameric complex in which MD-1 and MD-2 interact [8].

The structural and functional characterization of MD-1 and MD-2 proteins (MD proteins) has been delayed by the difficulties in producing active monomeric proteins. Indeed, MD proteins tend to aggregate in unstructured multimers containing intermolecular disulfides instead of intramolecular ones [9–11]. However, human MD-2 (hMD-2) has successfully been expressed in *Pichia pastoris*, purified, crystallized and its crystal structure solved in two complexed forms [11]. The first was obtained without added ligand and the determination of the structure revealed the presence of fatty acid molecules in the hydrophobic ligand-binding cavity. The second form was obtained by addition of lipid IVa, a substructure of LPS, which is inactive in human. Another group coexpressed human and mouse variants of MD-2 and TLR4 in a baculovirus expression system and successfully determined their atomic structures [2,12].

In previous work, we and others have found that lipid IVa displayed species-specific activities. Although inactive in human, lipid IVa is an agonist in mouse and horse [13–16]. We then established which residues were responsible for lipid IVa activity by generating chimeric forms of human and horse TLR4 and MD-2 proteins. In the course of this study, we characterized a chimera of MD-2 (HE82–89), in which residues 82–89 from horse were introduced into the human protein. Interestingly, HE82–89 displayed a significant level of constitutive activity suggesting that the area of interest was involved in the ligand-induced conformational change [16]. Here, we pursue the study of this chimera, as well as horse MD-2 (eMD-2) and human MD-1 (hMD-1) using the *P. pastoris* expression system and hMD-2 as a positive control. In standard conditions, we found that expression failed to produce detectable amounts of monomeric proteins. We then decided to screen constructs, yeast strains, recombinant colonies, culture medium, induction conditions and additives in order to increase the fraction of monomers. We found that the presence of a range of protein-specific additives in the culture medium during induction and along purification was a good strategy to decrease the propensity of these proteins to aggregate via intercatenary disulfide bonding.

## Material and methods

### Construct design

The coding sequences for mature hMD-2, eMD-2, HE82–89, and hMD-1 without their N-terminal signal sequences were cloned into the *Pichia* expression plasmids pPIC9K and pPICZα (Invitrogen). Primers for each construct were designed to incorporate an N-terminal *Eco*RI restriction site, His6-tag, and TEV cleavage site upstream of the gene of interest, followed by a C-terminal stop sequence and *Not*I restriction site. Both pPIC9K and pPICZα constructs allow secretion of MD-1 and MD-2 variants, as they contain the secretion signal sequence from the *Saccharomyces cerevisiae* α factor prepro peptide upstream of the multiple cloning site.

### *P. pastoris* strains

GS115 (*his4* genotype; Mut^+^, His^−^ phenotype) and KM71H (*arg4 aox1::ARG4* genotype; Mut^S^, His^+^, Arg^+^ phenotype) *P. pastoris* cells were used for screening protein expression in different backgrounds of histidine auxotrophy and alcohol oxidase activity. Mut^+^ stands for methanol-utilization-fast; Mut^S^, methanol-utilization-slow, which is the phenotype of cells lacking the AOX1 gene. His^−^ phenotype is caused by a *his4* mutation introduced in the histidinol dehydrogenase gene, which prevents the cells from synthesizing histidine. GS115 was therefore grown on complex medium or on minimal medium supplemented with histidine. His^+^ phenotype was restored in GS115 upon pPIC9K but not pPICZα transformation. Growth on minimal medium in absence of histidine was used as a selectable marker to isolate recombinant colonies upon pPIC9K. KM71H has an intact HIS4 gene but an *arg4* mutation in the argininosuccinate lyase gene and a wild-type ARG4 gene replacing the AOX1 gene resulting in a Mut^S^, Arg^+^ strain.

### Generation of recombinant *P. pastoris* strains

**Fig. 1:**
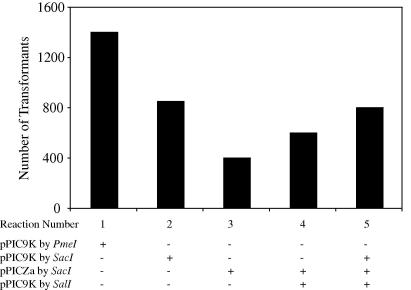
Optimal plasmid fragments for *Pichia* transformation. Several combinations of linearized plasmids were transformed into *Pichia* strain GS115. pPIC9K linearized at its 5′*AOX1* (with *SacI* and *PmeI*) or *HIS4* locus (with *SalI*) were transformed, along with pPICZα linearized at its 5′*AOX1* locus (with *SacI*). Transformation reaction 1: pPIC9K blunt ends at *5′AOX1* with *PmeI*; reaction 2: pPIC9K sticky ends at *5′AOX1* with *SacI*; reaction 3: pPICZα sticky ends at 5′AOX1 with *SacI*; reaction 4: double selection with pPIC9K sticky ends at *HIS4* with *SalI* and pPICZα sticky ends at *5′AOX1* with *SacI*; reaction 5: triple selection with pPIC9K sticky ends at *HIS4* with *SalI* and both pPIC9K and pPICZα with sticky ends at their respective *5′AOX1* loci with *SacI*. The number of antibiotic-resistant colonies isolated after transformation of each combination of linearized plasmids was recorded.

GS115 and KM71H cells were maintained and transformed according to the *Pichia* expression kit manuals (Invitrogen K1710-01, K1740-01 and K1750-01). The composition of the different *Pichia* culture media used in this study is described in Supplementary Table 1. Briefly, cultures were grown in a complex medium of YPD (Yeast extract Peptone Dextrose) to an OD_600nm_ of 1.3–1.5. Cells were harvested and washed in ice-cold sterile water twice before resuspension in 1 ml ice-cold 1 M sorbitol. All constructs were linearized prior to transformation. The empty host vectors pPIC9K and pPICZ〈 were prepared for transformation in parallel, for use as a negative control for MD-1 and MD-2 expression. pPIC9K constructs were linearized by digestion with either *SacI*, *PmeI*, or *SalI* (New England Biolabs). pPICZ〈 constructs were linearized by digestion with *SacI* or *PmeI*. Digestions were performed for 3 h at 37 °C, and complete linearization was verified by agarose gel electrophoresis. Following linearization, construct DNA was purified by ethanol precipitation and redissolved in 5–10 μL sterile water. Eighty microliters of electrocompetent cells were gently combined with 20 μL DNA. The amount of DNA per transformation was 20 μg for a single DNA preparation (Fig. 1a, reaction number 1, 2 and 3) or 10 μg of each preparation with a mixture of DNAs (Fig. 1a, reaction number 4 and 5). After pulsing at 1.5 kV, 25 μF, 200 Ω, 1 ml of ice-cold sorbitol was added to the electroporation cuvette (Cell Projects). Aliquots of this suspension were spread on minimal dextrose (MD) and Regeneration Dextrose Base (RDB) medium plates for pPIC9K transformants and on YPDS plates containing 100 μg/ml Zeocin™ for pPICZα transformants. Colonies grew within 5 days at 30 °C. pPIC9K colonies were subsequently screened on YPD plates in the presence of increasing concentrations of Geneticin® (between 0.25 and 10.0 mg/ml). Frozen stocks of all colonies growing on either Zeocin™-containing plates or exhibiting resistance to 10 mg/ml Geneticin® were prepared for backup and used in expression trials.

### Determination of the methanol-utilization phenotype

The methanol-utilization phenotype of antibiotic-resistant GS115 colonies was verified, as there is a small probability that recombination with the *Pichia* genome can disrupt the *AOX1* locus. Each colony was individually picked and streaked on a minimal methanol (MM) plate and a minimal dextrose (MD) plate in a regular pattern in the presence of histidine as required (for pPICZα transformants). Untransformed *Pichia* strains were also plated to serve as controls for the MutP^+^P and MutP^s^P phenotype, respectively. After incubation at 30 °C for 2 days, the plates were scored for growth by counting the number of colonies per plate.

### Recombinant protein production

A single colony was used to inoculate 5 ml YPD starter culture, and grown overnight shaking at 30 °C (225 rpm). This subculture was used to inoculate cultures either in minimal glycerol (MGY), buffered minimal glycerol (BMG), or buffered glycerol-complex (BMGY) medium. At an OD_600nm_ of 2–6, the cells were harvested by centrifugation at 1500–3000*g* for 5–10 min at room temperature and resuspended to an OD_600nm_ of 1.0 in minimal methanol (MM), buffered minimal methanol (BMM), or buffered methanol-complex (BMMY) medium to induce expression. The culture medium was combined in a sterile manner with additives filtered on 0.2 μm PES membrane when required. Methanol was added every 12–24 h to a final concentration of 0.5–2% to maintain induction. Cultures were carried out either in 24-well plates (with 2 ml per well), in 50 ml conical tubes (with 5 ml culture) placed at a 45° angle, 250 ml baffled flasks (with 25 ml culture) or in 2 L baffled flasks (with 200 ml culture) covered in 3 layers of sterile muslin gauze for optimal aeration. Aliquots were taken daily over a maximum period of 6 days to determine recombinant protein production by dot blot analysis and by SDS–PAGE and Western blotting on culture supernatants.

### Purification of secreted MD proteins

Recombinant His-tagged proteins were purified from the culture medium by metal affinity chromatography on a 5 ml Chelating HiTrap (GE Healthcare Life Sciences) after prefiltration on Sartoban P filters (Sartorius) and buffer-exchange of the culture supernatant by tangential flow filtration (Pall Corporation). These extra steps were required to dilute the ingredients in the culture medium that would strip the metal ions off the affinity column. Akta FPLC (Pharmacia) was used to elute the protein from the nickel column, in either gradient or step elution. The optimal elution scheme was found to be a step elution from Buffer A (50 mM Tris–HCl, 5 mM imidazole, pH 7.0) to Buffer B (50 mM sodium acetate, pH 3.5) in the presence of the appropriate anti-aggregation additive. Further purification was carried out using size-exclusion chromatography on HiLoad 16/60 Superdex 200 prep grade gel-filtration columns (GE Healthcare Life Sciences) in 50 mM sodium acetate, pH 5.0, also in the presence of the appropriate anti-aggregation additive. Purified proteins were analyzed by SDS–PAGE, Western and dot blotting.

### Western and dot blot analysis

Protein samples were separated by SDS–PAGE for Western blotting. Electroblotting to Hybond-C nitrocellulose membrane (Amersham Biosciences) was performed at 100 V for 45 min in Tris–Glycine transfer buffer. The membrane was reversibly stained with Ponceau S (Sigma) to ensure complete protein transfer. For dot blot analysis, 5 μl of each protein sample was added directly to Hybond-C nitrocellulose membrane (GE Healthcare Life Sciences), and allowed to dry completely. Membranes were blocked overnight at 4 °C in PBS, 0.05% Tween, 3% milk solution. After incubation with shaking for 1 h with primary anti-His antibody (BD Biosciences) at a dilution of 1:2500 in PBS-Tween-milk solution, membranes were washed three times for 15 min in PBS, 0.05% Tween. They were then incubated with shaking for 1 h with secondary HRP-conjugated-anti-mouse antibody (Sigma) at a dilution of 1:3000 in PBS-Tween-milk solution, and washed three times for 15 min in PBS-Tween. Chemoluminescent ECL substrate (Thermo Scientific) was added to the membrane, which was then exposed to X-ray film for 1 min for development.

## Results

### The expression vector, the site of linearization and the locus of integration used affect the yield of *P. pastoris* transformation

Human (hMD-2), equine (eMD-2) and a chimera of human MD-2, in which residues 82–89 where substituted with the equivalent equine sequence (HE82–89), as well as human MD-1 (hMD-1) were cloned downstream of an N-terminal His_6_-tag and a TEV cleavage site into the *P. pastoris* expression vectors pPIC9K and pPICZα Invitrogen to yield 8 constructs for yeast transformation. Before transformation, each construct was linearized by restriction enzyme digestion in order to stimulate recombination with the *Pichia* genome. The plasmids contain numerous suitable restriction sites at the 5′*AOX1* locus for both pPIC9K and pPICZα and at the *HIS4* locus for pPIC9K. To our knowledge, there was no previous study that had compared the efficiency of transformation of a given gene using (i) two expression vectors (ii) linearized at different sites (iii) for recombination at different loci (iv) transformed independently or (v) simultaneously. We therefore performed multiple reactions on one of the genes of interest (hMD-1) to check whether these parameters would affect the yield of transformation.

Recombinant pPIC9K was linearized at either the 5′*AOX1* site with *Sac*I or *Pme*I or the *HIS4* locus with *Sal*I. Recombinant pPICZα was cleaved at the 5′*AOX1* locus with *Sac*I. *Sac*I and *Sal*I generate sticky-ended fragments upon digestion, while *Pme*I generates blunt-ended fragments, which might influence the rate of recombination. Several combinations of these linearized fragments were transformed into *Pichia* strain GS115, and the number of transformants isolated after each transformation was recorded (Fig. 1). Histidine prototrophy followed by Geneticin®-resistance were used for selecting pPIC9K transformants in absence and in the presence of pPICZα whereas Zeocin™-resistance allowed the selection of pPICZα transformants.

Contrary to expectations the combined use of expression vectors did not improve the yield of transformation regardless of the site of integration (at the *AOX1* and the *HIS4* loci). Transformation of blunt-ended pPIC9K (*PmeI-*restricted) yielded almost twice as many colonies as transformation of sticky-ended pPIC9K plasmid (*SacI*-digested). This is expected considering the length of the homologous regions available for recombination, which is only 208 bp on the 5′ side of *AOX1* of the *SacI* cleavage site in comparison to 413 bp for *PmeI* out of a possible 942 bp at the *5′AOX1* promoter. Given these variations in size, we cannot conclude on the influence of blunt *versus* sticky-end DNA overhangs in the efficiency of recombination. In our hands, recombination seemed to be more dependent on fragment length than on the nature of the DNA ends at the 5′*AOX1* locus.

Furthermore we noticed that transformation with pPICZα yielded 70% fewer colonies than pPIC9K when both plasmids were linearized at the *SacI* site of *5′AOX1*. Also pPIC9K recombination at the *AOX1* site appeared to be favored compared to the *HIS4* locus according to the number of antibiotic-resistant colonies after transformation. This effect cannot be explained by the length of the homologous regions, which are 1199 bp at *HIS4* linearized by *SalI* (out of a total of 2535 bp) but is most likely a locus-dependent effect.

### The phenotypic background affects protein expression

By using pPIC9K and pPICZα vectors in GS115 and in KM71H strains, three different phenotypic backgrounds can be generated assuming that gene replacement does not occur. Upon transformation of GS115 (Mut^+^, His^−^) with pPIC9K the recombinant strain is Mut^+^, His^+^ whereas the use of pPICZα retains the Mut^+^, His^−^ phenotype and thus requires minimal medium to be supplemented with histidine. Transformation of KM71H (Mut^S^, His^+^) stays Mut^S^ regardless of the expression vector used. We have not used the wild type strain X33 (Mut^+^, His^+^) in this study as we can explore the same phenotype using GS115 and pPIC9K.

The methanol-utilization phenotype of GS115 colonies was verified (see Material and methods), as there is a small probability that recombination with the *Pichia* genome can disrupt the *AOX1* locus. All recombinant GS115 colonies were found to preserve the MutP^+^ phenotype according to their growth rate on minimum methanol (MM) compared to minimum dextrose (MD) plates. Screening for the methanol-utilization phenotype of KM71H colonies was not performed as the *AOX1* locus is inherently disrupted in this strain, and cannot be restored through recombination.

**Fig. 2:**
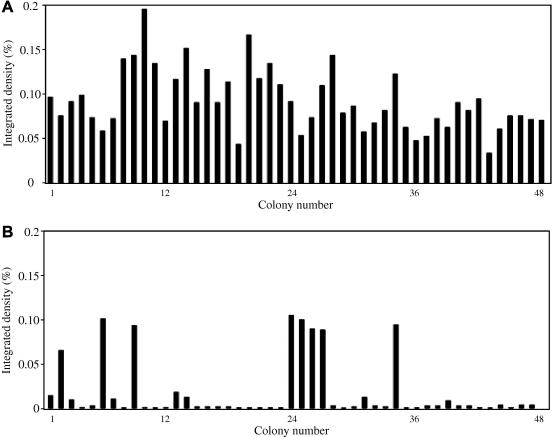
Effect of His phenotype on protein expression. (A) 48 colonies derived from transformation reaction 1 (His^+^ phenotype) were tested for expression levels in small-scale trials. (B) 48 colonies derived from transformation reaction 3 (His^−^ phenotype) were tested for expression levels in small-scale trials. The supernatant from each colony was analyzed by dot blot, and each signal was quantified as an integrated density percent of the total signal using ImageJ®.

As different phenotypes can have different advantages for protein expression we have compared the expression levels of hMD-1 produced in a His^+^ background such as the one obtained by using GS115–pPIC9K strains and in the His^-^ background of GS115–pPICZα (Fig. 2). 48 colonies isolated after transformation reaction **1** and **3** were selected for small-scale expression trials, and the relative expression levels were assessed by dot blot analysis of 5 μl culture supernatants. The signal given by each dot was quantified using the image-processing program, ImageJ® and compared in Fig. 2.

We found that only about 30% of the His^−^ GS115–pPICZα strains provided detectable protein expression in contrast to all 48 His^+^ pPIC9K transformants tested. It is not entirely clear if this is solely due to the phenotype. We cannot rule out that the double selection of pPIC9K transformants (histidine prototrophy and Geneticin®-resistance) and the number of copies integrated in the *Pichia* genome, are also at play, as we have not characterized these colonies further.

Based on the number of transformants, as well as the relative expression levels of colonies we found that pPIC9K linearized by *PmeI* at the *5′AOX1* locus was the best expression vector in our hands and therefore employed for all subsequent transformation reactions into *P. pastoris.* Consequently, we only examined Mut^+^ or Mut^S^ backgrounds on the effect of protein expression in the rest of this study by using either GS115 or KM71H pPIC9K-modified strains.

### Single colony screening reveals a difference of up to 20-fold in the yield of MD variant protein secretion

First, the sensitivity and the specificity of anti-His-tag blots were assessed as follows. Increasing amounts of purified His-tagged protein (TEV protease) were blotted and a strong signal was observed at a concentration of at least 1 mg/L, which represents approximately 50 nM for a 20 kDa His-tagged protein (Supplementary Fig. 1). The absence of background signal was checked by blotting culture supernatants of antibiotic-resistant *Pichia* strains transformed with a mixture of *PmeI*-linearized non-recombinant pPIC9K and pPICZα vectors and induced under the same conditions as those used for MD protein expression (Supplementary Fig. 2). These controls validate the use of Western and dot blot in this study.

**Fig. 3:**
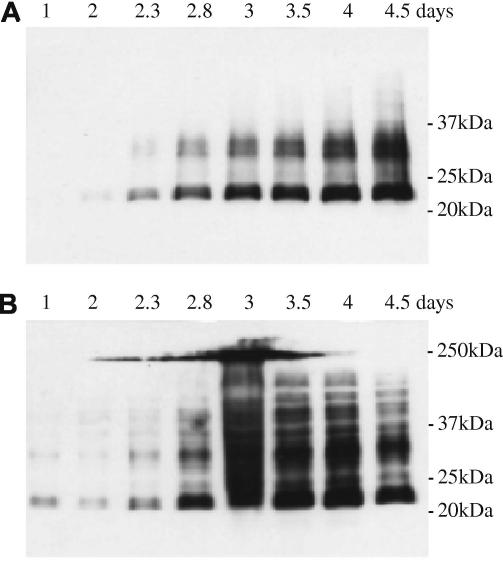
Time course of expression. A typical time course of expression was performed on a single colony for each recombinant *Pichia* strain. Samples were taken at various time points throughout expression over a week and analyzed by anti-His Western blot in reducing conditions. Supernatants (A) and cell lysates (B) from GS115/hMD-2 cultures are represented here.

Next, a time course of expression was performed on a single colony for each recombinant *Pichia* strain. Samples were taken at various time points throughout expression over a week and analyzed by Western blotting in reducing conditions. Expression of all four proteins appeared to plateau after 72 h in Mut^+^ GS115 (Fig. 3) and after 96 h in Mut^S^ KM71H (not shown).

**Fig. 4:**
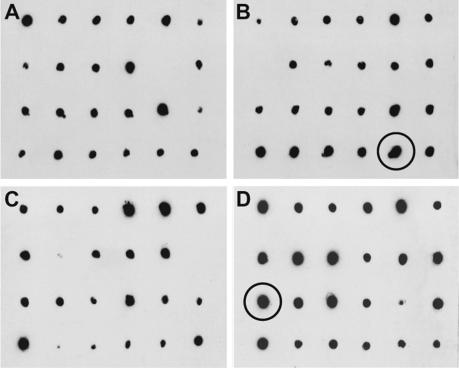
Single colony screening for the detection of the highest-expressing colony. 48 recombinant colonies of strain GS115 and KM71H expressing each variant were induced in small-scale expression screens, and the relative expression level of each colony was compared by anti-His dot blotting. Colonies 1–24 (A) and 25–48 (B) expressing an MD variant in GS115 were analyzed, along with colonies 1–24 (C) and 25–48 (D) expressing the same MD variant in KM71H. The highest-expressing colony of each strain, which was selected for all future expression studies, is circled. The MD variant shown here is HE82–89.

Then, 48 recombinant colonies were screened by dot blotting for their relative expression levels for each MD protein at the optimal time determined for each strain (72 h for GS115 and 96 h for KM71H) (Fig. 4). Significant variations in expression levels were observed among colonies. Selection of the highest-expressing colony improved the yield of subsequent expression studies as much as 20-fold.

### Optimal culture conditions vary according to protein

**Fig. 5:**
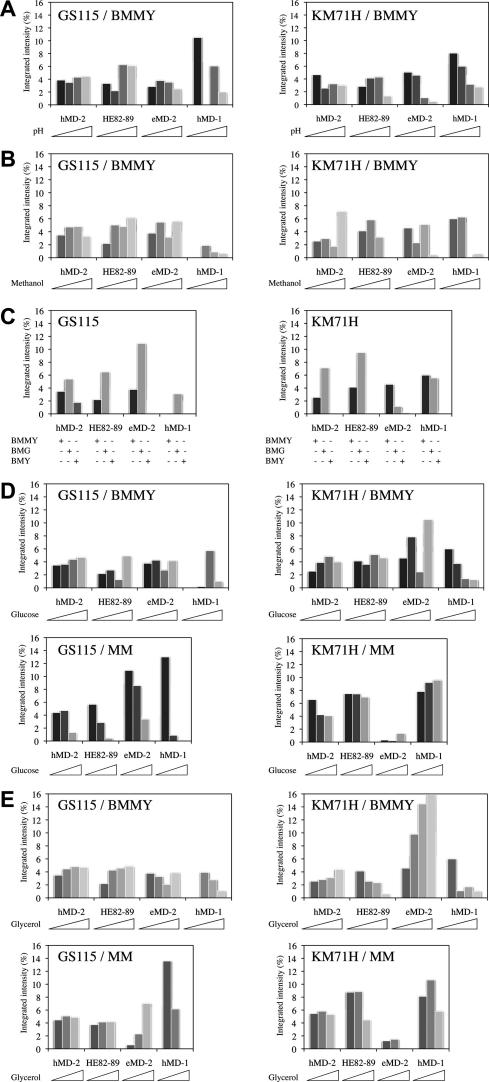
Optimization of induction. The highest-expressing colony of each protein in each strain was expressed in 24 different culture conditions (see Supplementary Table 1 for culture medium composition), on a small scale, and supernatants were analyzed by dot blotting followed by signal quantification with ImageJ®. The relative expression in each condition is indicated by the integrated density percent of the total signal present for proteins expressed in *Pichia* strain GS115 (left panels) and KM71H (right panels). (A) Effect of pH. Values between 5.0 and 8.0 have been tested in BMMY in one unit increments. (B) Effect of methanol. Daily addition of increasing concentrations (0.5%; 1.0%; 1.5%; 2.0%) of methanol have been tested. (C) Effect of yeast extract and peptone. Complex BMMY and minimal buffered media in absence and in the presence of 1% glycerol, BMG and BMM, respectively, have been compared here. (D) Effect of glucose. Increasing concentrations (0%; 0.1%; 0.2%; 0.5%) of glucose have been tested in either BMMY or MM for each construct as indicated. (E) Effect of glycerol. Increasing concentrations (0%; 0.1%; 0.2%; 0.5%) of glycerol have been tested in either BMMY or MM on each construct.

In order to further improve the yield from *Pichia* expression, the highest-expressing colony of each protein in each strain was subjected to a small-scale screen of varying media conditions during protein expression (Fig. 5). Culture supernatants from expression in each condition were analyzed by dot blotting, followed by signal quantification. Because samples of equal volumes (5 μl) were analyzed without OD_600nm_ readings for normalization, this assay can only suggest trends in expression that are not quantitative. The optimal media type appeared to vary according to protein, although hMD-2 and HE82–89 exhibited overall similar behavior, which is expected due to their nearly identical amino acid sequences.

### The effect of pH

The recombinant proteins used in this study cover a large range of isoelectric points with hMD-2 being the most basic and hMD-1 the most acidic (p*I*_hMD-2_ = 8.34; p*I*_HE82-89_ = 8.15; p*I*_eMD-2_ = 7.23; p*I*_hMD-1_ = 5.50). Therefore we decided to test BMMY media buffered between pH 5.0 and 8.5 (Fig. 5A) as well as unbuffered minimal medium MM, the pH of which drops below 3.0 during induction (Fig. 5D and E). We found that the pH did impact the expression levels of MD proteins. The observed trend suggests that the optimal pH for expression in buffered complex media peaks around each protein’s p*I*. Nevertheless protein expression levels were generally highest in unbuffered minimal media.

### The effect of methanol

Although a daily addition of 0.5% methanol is recommended for induction (Invitrogen manuals), some studies have optimal yields attained at higher concentrations [17,18]. Thus, 0.5%, 1%, 1.5%, and 2% methanol were screened for their effect on expression (Fig. 5B). In our hands, there was no obvious trend using higher methanol concentrations, which was therefore kept at 0.5%. Although methanol was supplemented daily initially, we subsequently opted for addition every 12 h for a more sustained expression.

### The effect of culture media composition

We tested protein secretion into three different media BMMY, BMG and BMM all buffered at pH 6.0 (Fig. 5C) with expression induced by daily addition of 0.5% methanol. BMMY is a complex medium and differs from minimal BMG and BMM media by its content in yeast extract and peptone. BMG contains 1% glycerol, which is thought to repress *AOX1*-driven gene expression. We found that all four MD proteins shared the same feature of media preferences and exhibited extremely reduced expression levels in buffered minimal medium BMM.

### The effect of alternative carbon sources

Even though high concentrations of alternative carbon sources including glycerol and glucose depresses the induction mechanism via *AOX1*, small amounts of glycerol and glucose have been shown to improve the yield from *Pichia* expression [19,20]. Given our previous result with BMG (Fig. 5C), 0.1%, 0.2%, 0.5% glucose (Fig. 5D) and glycerol (Fig. 5E) were screened for their effect on yield as well. Screening was performed in both minimal medium MM and buffered complex medium BMMY pH 6.0, as there was no rationale for expecting their effect on expression to be the same in both media types. We observed that the presence of at least 0.1% of an alternative carbon source, either glucose or glycerol, appeared to increase expression levels.

### The effect of Mut phenotype

We compared Mut^+^ or Mut^S^ backgrounds on the effect of protein expression by using either GS115 or KM71H pPIC9K-modified strains. The only detectable strain-specific media preference was an even larger increase in yield associated with the inclusion of small amounts of glucose or glycerol in the medium of KM71H expression cultures. The optimal expression conditions determined for each strain were as follows: for hMD-2, minimal medium MM + 0.2% glycerol for GS115 and MM + 0.1% glycerol for KM71H; for HE82–89, we used BMMY at pH 7.0 for GS115 and MM + 0.2% glycerol for KM71H; for eMD-2, it was MM + 0.1% glucose for GS115 and BMMY + 0.2% glycerol for KM71H; for hMD-1, we chose MM + 0.1% glycerol for GS115 and MM + 0.2% glycerol for KM71H. This resulted in at least a 2-fold improvement of yield over standard BMMY induction medium upon quantification via large-scale expression and purification trials.

### MD-1 and MD-2 variants are produced as a heterogeneous collection of oligomers

In contrast to the variation in the yield of protein production, no colony-specific variation was observed in the oligomeric state of MD-1 and MD-2 variants (data not shown). Nickel purified protein was submitted to size-exclusion chromatography and eluted in the void volume of the gel-filtration column (Fig. 7). Non-reducing SDS–PAGE revealed the presence of “monomers” and multimeric species in the gel-filtration peak fractions and suggested that aggregation was mediated via both intermolecular disulfide bonds and non-covalent protein–protein interactions.

### The effect of culture additives on MD proteins

Additives are traditionally assessed on proteins in solution for their effect on folding and solubility. Some of them can be classified as kosmotropes or chaotropes according to their effect on the stability and the structure of water, which affects interactions between solvent and proteins as well as protein–protein contacts. These effects are described in the Hofmeister or lyotropic series. Additives are not routinely tested in yeast cultures. Since aggregation seemed to occur during culture for MD proteins, we reasoned that additives might have a stabilizing effect on the secreted proteins. However, these agents may also affect *Pichia* growth, cell metabolism, and protein biosynthesis kinetics if they are taken up into the cells and used as nutrients or cofactors. The effect of additives in cell cultures might therefore be direct or indirect and affect a range of cellular processes including recombinant protein production.

**Fig. 6:**
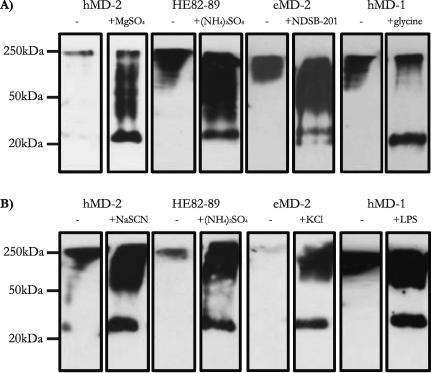
Effect of culture media additives on the prevention of intermolecular disulfide formation. A recombinant colony of each construct was induced in the absence of culture additives (−) and in the presence of a given anti-aggregation agent at the following concentrations: 0.2 M MgSO_4_; 0.15 M (NH_4_)_2_SO_4_; 0.5 M NDSB-201; 1.5% glycine; 0.1 M NaSCN; 0.5 M KCl; 0.01 mg/ml LPS from *E. coli* O127:B8 (Sigma L3129). Samples of supernatants harvested from each construct were analyzed by Western blotting in non-reducing conditions. (A) GS115 cultures and (B) KM71H cultures.

A panel of 30 different anti-aggregation agents was selected for inclusion in the induction culture medium (Supplementary Table 2). Small-scale screens were performed on each protein. The highest-expressing recombinant GS115 or KM71H colony was grown in 72 different conditions in 24-well plates containing different anti-aggregation additives in the induction media. The effect of each additive was tested in both buffered complex induction medium as well as minimal induction medium, as the different pH and composition of these two media might have differential effects upon the additives’ efficacy. After 72 h (GS115) or 96 h of induction (KM71H), supernatants were harvested and analyzed by dot blot (Supplementary Fig. 3–6). From this initial analysis it appeared that some additives were detrimental to the overall protein expression levels. These agents were excluded from any subsequent analysis. Others gave a strong signal on dot blot and were further analyzed by Western blot under non-reducing conditions in order to test their ability to prevent intermolecular disulfide bond formation (Supplementary Fig. 7–14). The best results are shown in Table 1 and Fig. 6.

### The presence of magnesium sulfate in culture inhibits hMD-2 aggregation

Without the inclusion of an additive, a small almost undetectable proportion of MD-2 is expressed in its monomeric form (Fig. 6). Overall, the yield of hMD-2 expression in both GS115 and KM71H strains benefits from most detergents and kosmotropic agents, as well as chaotropic additives and the MD-2 binding ligand LPS. In particular, magnesium sulfate has a dramatic effect upon expression of hMD-2 in GS115. Both magnesium sulfate and magnesium chloride improve production in KM71H cells. Magnesium salts do not only possess kosmotropic properties that can benefit protein folding and solubility they are also taken up by cells and affect intracellular processes. Interestingly the effect of magnesium salts is strongest in minimal induction medium, in which nutrients and cofactors might be a limiting factor for proper protein production. Several other additives with unrelated chemical properties appear to increase the expression of monomeric MD-2 when expressed in KM71H, including NaSCN, NDSB-201, and LPS itself.

### The presence of ammonium sulfate in culture inhibits HE82–89 aggregation

Inclusion of additives in the culture medium also enriched the HE82–89 population for its monomeric form. However, ammonium sulfate, rather than magnesium sulfate, exhibited the most dramatic effect in encouraging monomer formation. Ammonium sulfate is both a kosmotropic agent and a nutrient that might alter cell metabolism. In comparison with the effect of magnesium salts on hMD-2, ammonium salts were only effective on the mutant protein in complex medium. As a slightly weaker kosmotropic agent compared to magnesium sulfate, it would also weaken the entropic cost of hydrating hydrophobic patches, reducing water structure in the process. The preferred effect of ammonium sulfate in the case of HE82–89 may reflect the subtle difference in amino acid sequence between this mutant and hMD-2 and was observed in both yeast strains. Out of the four MD proteins tested, HE82–89 depended the least on the presence of LPS for monomer production as expected for a constitutively active protein. In contrast, the effect of the amino acid additive glycine exhibited a particularly dramatic effect upon expression of the mutant in KM71H, increasing both the yield and proportion of monomers formed.

### A non-detergent sulfobetaine for eMD-2

The additive, which caused the most dramatic inhibition of intermolecular disulfide bond formation was the non-detergent sulfobetaine NDSB-201 for eMD-2 (Fig. 6). This additive significantly increased the yield of expression as well as the relative proportion of monomers in the total eMD-2 population. The weak kosmotropic salts NaSCN and KCl also inhibited intermolecular disulfide bond formation among eMD-2 species. Finally, LPS appeared to enrich the eMD-2 population for its monomeric form in buffered complex medium, but not in minimal medium, as observed for both hMD-2 and hMD-1.

### LPS, but not hMD-2, inhibits hMD-1 aggregation in culture

As seen for other MD proteins, expression of hMD-1 in the absence of additives resulted in a limited proportion of monomeric hMD-1 in culture supernatant (Supplementary Fig. 13–14). However, this proportion was significantly enhanced by additives such as LPS, NDSB-201, the reducing agent l-oxidized glutathione, and chloride-containing salts. It is often found that inclusion of a ligand and/or coexpression of a physiological partner can stabilize a protein that would otherwise be unstable or unfolded. In order to test this hypothesis, we compared hMD-1 expression and aggregation levels in the presence or absence of LPS and hMD-2. Interestingly, inclusion of LPS or fetal calf serum, but not hMD-2, partially prevented aggregation in either strain GS115 or KM71H and only in pH 6.0-buffered complex medium. Both hMD-1 and hMD-2 produced in KM71H strains appear to be more susceptible to the disruption of intermolecular disulfide bonds through anti-aggregation agent interference. The amino acid glycine and the non-detergent sulfobetaine NDSB-201 caused a dramatic enhancement in the proportion of hMD-1 which does not form intermolecular disulfide bonds, with glycine exhibiting a slightly stronger effect.

### Quantitative evaluation of the effect of additives

**Fig. 7:**
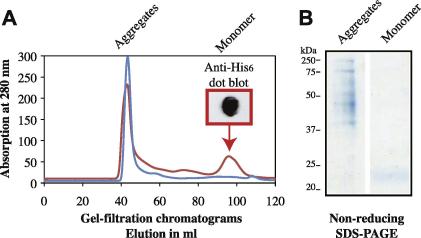
Inclusion of additives in culture medium and purification buffers allows isolation of monomeric protein. Typically, expression cultures were prepared, and the harvested supernatant was concentrated and buffer-exchanged by tangential flow filtration prior to protein purification. After Nickel affinity chromatography, the sample was concentrated, and purified by gel filtration on a HiLoad 16/60 Superdex 200 column. (A) In absence of additive proteins from an 8L-culture of GS115–hMD-2 in BMMY medium eluted in the void volume only (blue chromatogram; aggregates eluted at 43.3 ml). Optimization of the culture medium (MM + 0.2% glycerol instead of BMMY) and inclusion of 0.2 M MgSO_4_ significantly improved the yield of monomeric hMD-2 protein production (red chromatogram; aggregates eluted at 42.9 ml and monomers at 96.8 ml). The nature of the second peak was confirmed by anti-His dot blotting. (B) Gel-filtered proteins taken from both peak fractions with elution profiles corresponding to aggregates and monomeric proteins were analyzed on a non-reducing Coomassie-stained SDS–PAGE gel.

Whereas small-scale screening only allowed qualitative evaluation of the effect of additives, large-scale preparations were required for a quantitative assessment. Only the additives predicted to have the strongest effect on each protein were analyzed. In order to estimate the effect of each additive, cultures were grown in either complex or minimal medium with and without additive. Secreted proteins were prepped in a two-step purification protocol described in Material and methods. Typically several liters (up to 8 L) of expression cultures were prepared, and the harvested supernatant was concentrated and buffer-exchanged by tangential flow filtration prior to protein purification. After Nickel affinity chromatography, the sample was concentrated, and submitted to gel filtration on a HiLoad 16/60 Superdex 200 column (Fig. 7). In the absence of additives MD proteins eluted in the void volume. Inclusion of the appropriate additive in the culture medium and the purification buffers significantly improved the yield of monomeric protein production. The best result obtained with hMD-2 was in the presence of 0.2 M MgSO_4_ with the production of approximately 15% monomeric protein out of 4 mg total protein subjected to gel-filtration.

## Discussion

It has been conclusively proven that monomeric MD-2 is the active species responsible for LPS binding and TLR4 activation. However, the oligomeric form of secreted MD-2 has been reported in multiple expression systems, including mammalian HEK293 and HeLa cells [10,21–24]. It remains unknown whether this oligomeric form exists physiologically or whether it is an artifact of overexpression. This study demonstrates another expression system in which the disulfide-mediated oligomerization of MD-2 as well as MD-1 proteins is observed. It is unclear how misfolded proteins interlinked into high molecular weight oligomers escape the quality control mechanisms which prevent improperly folded proteins from leaving the ER. MD proteins may be properly folded in the ER, passing through the quality control mechanisms normally, and may oxidize and aggregate through covalent and non-covalent interactions extracellularly. This hypothesis is plausible because the secretory pathway provides chaperones and a protective environment for newly folded proteins, whereas the extracellular medium affords contact with oxidative molecules near the medium-air interface, which may oxidize MD proteins and mediate aggregation. On the other hand, Western blotting in non-reducing conditions in this study revealed the same array of oligomeric species present in both the lysate and the supernatant of *Pichia* induction cultures. Also, a previous study of intracellular *Pichia* expression observed the same range of oligomeric species with a cytoplasmic expression system [25]. Thus, aggregation and oligomerization mediated by improper folding and disulfide bond formation may occur as the protein is synthesized. This explanation is not implausible, as misfolded MD proteins are known to be able to escape the quality control mechanism in mammalian cells too: mutated MD-2 lacking all seven cysteine residues is secreted into the medium in HEK293 [9]. Thus, MD-2 may form intermolecular disulfide bonds and oligomeric aggregates early in its folding process, and may evade quality control of *Pichia* by an unknown mechanism.

In this study, a novel method was developed which provides a means of stabilizing secreted proteins and inhibiting improper formation of intermolecular disulfide bonds during secreted protein expression. Due to the heterogeneity and instability of MD proteins expressed in this system, this method was required to isolate monomeric protein from *Pichia* culture supernatants. The mechanism by which this novel method decreases aggregation and the formation of intermolecular disulfide bonds is not entirely straightforward. *Pichia* cells can absorb anti-aggregation agents such as magnesium sulfate. The intracellular effects of such an additive can then either be direct or indirect. Magnesium sulfate could help solubilize newly synthesized proteins intracellularly as well as extracellularly, but it could also affect protein production as a nutrient and/or a cofactor affecting a variety of processes including cellular metabolism, protein synthesis and molecular transport. Alternatively, many of the anti-aggregation agents probably stabilize secreted protein in the extracellular milieu, preventing oxidation of misfolded species and aggregation via hydrophobic and ionic interactions. In keeping with the effect of additives in solution, we also found that pH affected protein expression. pH can affect their solubility via its effect on charged and polar surface residues that interact with ionic groups in the solvent. A protein may precipitate out of solution in absence of such ionic interactions. All MD proteins behaved well in the acidic environment of unbuffered minimal medium, which can prevent oxidation of free sulfhydryl side-chains. Surprisingly, the optimal pH for expression in buffered complex medium peaked around each protein’s p*I*. This is an unusual effect unless there is a discrepancy between the theoretical p*I* and the real electrostatic charge of the molecule. The tested kosmotropic and chaotropic agents likely do not interact with the protein directly, but rather affect the ordering of water molecules around the protein, raising or lowering the entropic cost of hydrating its hydrophobic regions. Nutrients and cofactors potentially alter cell metabolism and protein synthesis kinetics. This effect was generally more pronounced in minimal medium, compared to complex medium, in which these components might be found in limited amounts. The detergents tested may interact with the protein, in particular at the exposed hydrophobic regions and at the site of LPS binding. The mild reducing agents tested likely stabilize the free sulhydryl groups and prevent their oxidation in the extracellular medium. The ligand tested most likely exerts its stabilizing effect through direct binding.

Regardless of the mechanism of action at play, this novel method for inhibiting protein aggregation may be relevant for the preparation of any secreted glycoprotein, which exhibits similar instability. There is some precedent for the unusual tendency to aggregate by intermolecular disulfide bond formation in culture supernatants. For example, secreted meprin A metalloproteinase produced in HEK293 cells forms higher-order molecular weight oligomers when secreted into the medium [26]. Similarly, Norrie disease protein (Norrin) forms disulfide-linked oligomers when expressed by mammalian COS-7 cells [27]. A common feature of all proteins, which exhibit this unusual behavior upon secretion is the presence of N-linked glycosylation sites. Interestingly, one group has found that mutation of the N-linked glycosylation sites of meprin A metalloproteinase abolished inter-subunit disulfide bond formation [28]. Glycosylation affects protein properties such as solubility and folding, and thus it would be interesting to analyze if mutation of the N-linked glycosylation sites of MD-2, which has been conducted in previous studies, might reduce the inter-subunit disulfide bond formation among MD-2 species in various expression systems.

Finally the effect of LPS on the production of monomeric MD-1 is surprising as MD-1 was long thought not to bind LPS [29]. The solubilizing effect of LPS can only be explained by an interaction with MD-1, since LPS itself possesses no kosmotropic or chaotropic properties which might affect the oligomeric state of the protein. During the editing of this manuscript, two crystal structures of MD-1 have been published elsewhere [30,31]. These studies on mouse and chicken MD-1 were supplemented with biochemical analysis that also supports LPS binding. The functional importance of MD-1 needs to be reassessed in the light of these recent findings. However, this property allows it to play an active role in modulating bacterial infection and LPS signaling.

## Figures and Tables

**Table 1:** Optimal additives for production of monomeric MD protein. A panel of anti-aggregation additives including kosmotropic agents, chaotropic agents, amino acids, ionic detergents, non-ionic detergents, zwitterionic detergents, reducing agents, and others were selected (Supplementary Table 2). Each anti-aggregation additive was tested in small-scale cultures both in buffered complex induction medium (BMMY) as well as minimal medium (MM), as the different pH and composition of these media influenced the effect of these agents upon the inhibition of protein aggregation. First, each agent was tested for its ability to promote or not to interfere with secretion of MD proteins by dot blot analysis (Supplementary Fig. 3-6). Then a subset of the additives that had given a strong expression signal was further characterized by Western blot analysis in non-reducing conditions (Supplementary Fig. 7–14). The agents were evaluated for their potency to inhibit intercatenary disulfide bonding and optimal conditions are summarized here.

Proteins	*Pichia pastoris* strains	
**GS115**	**KM71H**
hMD-2	MM + 0.2M MgSO_4_; MM + 1% FCS;	MM + 0.1M MgCl_2_; MM + 0.2M MgSO_4_; MM + 0.01mg/ml LPS;
BMMY + 0.01mg/ml LPS; BMMY + 1% FCS;	BMMY + 0.1M NaSCN; BMMY + 0.01mg/ml LPS; BMMY + 0.5M NDSB-201;

HE82-89	WMVIII;	
BMMY + 0.15M (NH_4_)_2_SO_4_;	BMMY + 1.5% glycine; BMMY + 0.15M (NH_4_)_2_SO_4_;

eMD-2	MM + 10% FCS;	MM + 0.15M (NH_4_)_2_SO_4_;
BMMY + 0.01mg/ml LPS; BMMY + 0.5M NDSB-201;	BMMY + 0.1M NaSCN; BMMY + 0.5M KCl;

hMD-1		MM + 1% L-glutathione; MM + 0.01mg/ml LPS;
BMMY + 0.01mg/ml LPS; BMMY + 1.5% glycine; BMMY + 0.5M NDSB-201;	BMMY + 0.01mg/ml LPS; BMMY + 0.1M CaCl_2_; BMMY + 0.1M MgCl_2_; BMMY + 0.5M KCl.

## Appendix A Supplementary data

Supplementary data A document containing Figures and Tables.

## Footnotes

1 *Abbreviations used:* HE82-89, Human MD-2 chimera protein containing equine residues 82–89; LPS, lipopolysaccharide; MD proteins MD-1 and MD-2, Myeloid differentiation factors 1 and 2; NDSB, non-detergent sulfobetain; RP105, Radio Protective 105 kDa receptor; TLR4, Toll-like Receptor 4.
