# Supplementary material for: LPS ligand and culture additives improve production of monomeric MD-1 and 2 in Pichia pastoris by decreasing aggregation and intermolecular disulfide bonding
Source: Protein Expr Purif. 2011 Apr;76(2):173–83. doi: 10.1016/j.pep.2010.11.018 (PMC3032050; doi:10.1016/j.pep.2010.11.018)
Supplement: Supplementary data — A document containing Figures and Tables. [file mmc1.doc]

**LPS ligand and culture additives improve production of monomeric MD-1 and 2 in *Pichia pastoris* by decreasing aggregation and intermolecular disulfide bonding**

***Supplementary material***

**Supplementary Table 1.** Culture medium composition.

Components of liquid and solid media (agar plates require an additional 2 % agar) for *Pichia* phenotype verification and protein expression. Minimal plates were used to evaluate the methanol utilization phenotypes (Mut^+^ or Mut^S^) according to the manufacturer’s instructions (Invitrogen Cat. no. K1750-01 for pPIC9K transformants and Cat.no. K1740-01 for pPICZα transformants).

| **Medium** | **Components** | **Application** |
| --- | --- | --- |
| BMG | 1.34% yeast nitrogen base, 4 × 10^-5^ % biotin, 1% glycerol, 100mM potassium phosphate pH 6.0 | Buffered minimal medium containing glycerol repressing AOX1 gene expression |
| BMGY | 1.34% yeast nitrogen base, 4 × 10^-5^ % biotin, 1% glycerol, 100mM potassium phosphate pH 6.0, 1% yeast extract, 2% peptone | Buffered complex medium containing glycerol repressing AOX1 gene expression |
| BMM | 1.34% yeast nitrogen base, 4 × 10^-5^ % biotin, 0.5% methanol, 100mM potassium phosphate pH 6.0 | Buffered minimal medium containing methanol for protein secretion into a pH-controlled medium |
| BMMY | 1.34% yeast nitrogen base, 4 × 10^-5^ % biotin, 100mM potassium phosphate pH 6.0, 0.5% methanol, 1% yeast extract, 2% peptone | Buffered complex medium containing methanol for protein secretion into a pH-controlled medium |
| MD | 1.34% yeast nitrogen base, 4 × 10^-5^ % biotin, 2% dextrose | Minimal dextrose medium for His^+^ recombinant *Pichia* growth |
| MDH | 1.34% yeast nitrogen base, 4 × 10^-5^ % biotin, 2% dextrose, 0.04% histidine | Minimal dextrose medium for His^-^ recombinant *Pichia* growth |
| MGY | 1.34% yeast nitrogen base, 4 × 10^-5^ % biotin, 1% glycerol | Minimal medium containing glycerol repressing AOX gene expression |
| MM | 1.34% yeast nitrogen base, 4 × 10^-5^ % biotin, 0.5% methanol | Minimal methanol for His^+^ recombinant *Pichia* growth and protein expression |
| MMH | 1.34% yeast nitrogen base, 4 × 10^-5^ % biotin, 0.5% methanol, 0.04% histidine | Minimal methanol for His^-^ recombinant *Pichia* growth |
| RDB | 1M sorbitol 2% dextrose 1.34% yeast nitrogen base, 4 x 10^-5^ % biotin, 0.005% amino acids | Regeneration Dextrose Base for plating pPIC9K transformants |
| WMVIII | Sucrose 50 g/l, NH_4_H_2_PO_4_ 0.25 g/l, NH_4_Cl 2.8 g/l, sodium glutamate 10 g/l, MgCl_2_x6H_2_0 0.25g/l, CaCl_2_x2H_2_0 0.1g/l, KH_2_PO_4_ 2g/l, MgSO_4_ x7H_2_O 0.55 g/l, myo-inositol 75 mg/l, ZnSO_4_ x7H_2_0 1.75mg/l, FeSO_4_x2H_2_0 0.5 mg/l, CuSO_4_x5H_2_0 0.1 mg/l, MnCl_2_x 4H_2_0 0.1 mg/l, Na_2_MoO_4_x2H_2_0 0.1 mg/l, nicotinic acid 10mg/l, pyridoxin-HCl 25g/l, thiamine-HCl 10mg/l, biotin 2.5 mg/l, calcium pantothenate 50 mg/l, EDTA 10μM | Minimal medium without yeast nitrogen base |
| YPD | 1% yeast extract, 2% peptone, 2% dextrose | Rich complex broth used for general growth and storage of wild type and mutant His^-^, Arg^-^ *Pichia* strains |
| YPDS ± Zeocin™ | 1% yeast extract, 2% peptone, 2% dextrose, 1M sorbitol ± 100 μg/ml Zeocin™ | Rich complex broth used for selection of *Pichia* Zeocin™-resistant transformants |

**Supplementary Table 2.** Concentration and chemical property of additives used in this study.

| **Additive** | **Concentration** | **Additive Type** |
| --- | --- | --- |
| L-arginine | 0.4 M | amino acid |
| glycine | 1.5 % | amino acid |
| CaCl_2_ | 0.1 M | chaotrope |
| MgCl_2_ | 0.1 M | chaotrope |
| NaSCN | 0.1 M | chaotrope |
| Urea | 1 M | chaotrope |
| FCS | 1 – 10 % | Fetal calf serum |
| Li_2_SO_4_ | 0.1 M | kosmotrope |
| MgSO_4_ | 0.2 M | kosmotrope |
| (NH_4_)_2_SO_4_ | 0.15 M | kosmotrope |
| LPS | 0.01 mg/mL | ligand |
| NDSB-201 | 0.5 M | non-detergent sulfobetaine |
| DDM | 100 μM – 1 mM | non-ionic detergent |
| OGP | 50 mM | non-ionic detergent |
| Pluronic F-68 | 0.05 % | non-ionic detergent |
| Tween-20 | 60 μM | non-ionic detergent |
| TritonX-100 | 0.05 % | non-ionic detergent |
| Mineral oil | 0.05 % | paraffinic oil |
| DMF | 1 % | polar aprotic solvent |
| DMSO | 1 % | polar aprotic solvent |
| MEG | 20 % | polyhydric alcohol |
| sorbitol | 20 % | polyhydric alcohol |
| L-glutathione | 1 % | reducing agent |
| β-mercaptoethanol | 1 - 5 mM | reducing agent |
| sucrose | 0.5 M | sugar |
| KCl | 0.5 M | weak kosmotrope |
| K_2_CO_3_ | 0.1 M | weak kosmotrope |
| NaCl | 0.5 M | weak kosmotrope |
| Na_2_CO_3_ | 0.1 M | weak kosmotrope |
| CHAPS | 1 - 10 mM | zwitterionic detergent |

**
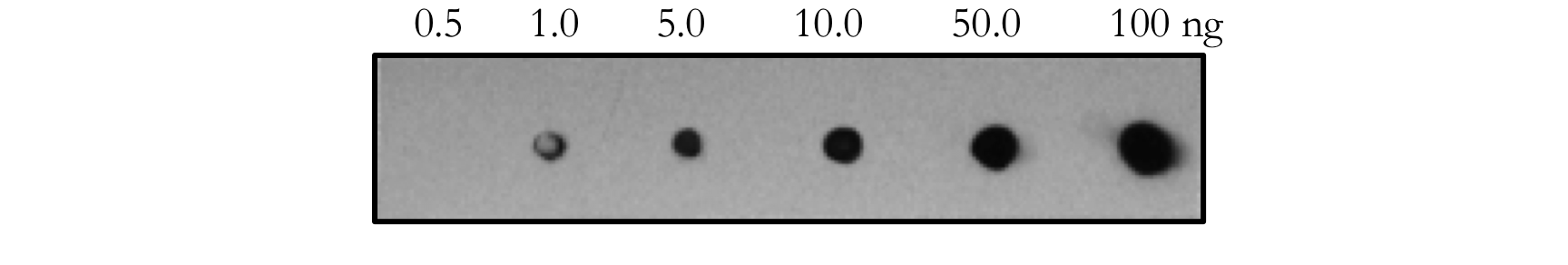
**

**Supplementary Figure 1: Sensitivity of anti-His blotting.** Increasing amounts of purified His_6_-tagged TEV protease (5 μl were deposited onto the membrane at concentrations between 0.1 and 20.0 ng/μl) were blotted as described in Material and Methods. A strong signal was observed in presence of a concentration of least 1 mg/l.

**
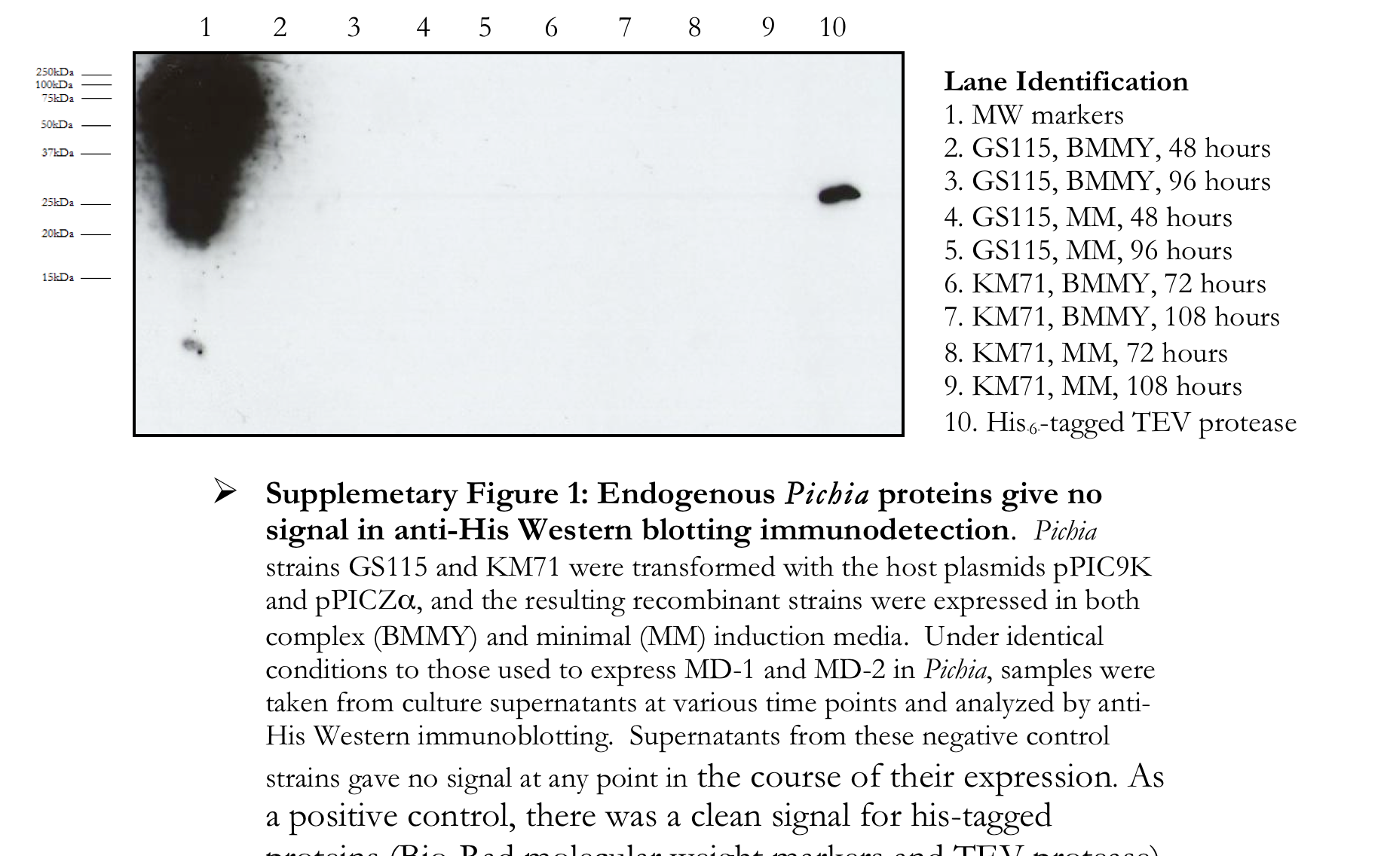
**

**Supplementary Figure 2: Endogenous *Pichia* proteins give no signal in anti-His Western blotting immunodetection**. *Pichia* strains GS115 and KM71H were transformed with the host plasmids pPIC9K and pPICZα, and the resulting recombinant strains were expressed in both complex (BMMY) and minimal (MM) induction media. Under identical conditions to those used to express MD-1 and MD-2 in *Pichia*, samples were taken from culture supernatants at various time points and analyzed by anti-His Western immunoblotting. Supernatants from these negative control strains gave no signal at any point in the course of their expression. His-tagged proteins were used as a positive control (Bio-Rad molecular weight (MW) markers and 20ng TEV protease (20 μl at 1 ng/μl), lane 1 and 10, respectively). These positive and negative controls validate the use of Western and dot blot in this study.

**Supplementary Figures 3-6: Screening of culture additives, step 1: dot blot analysis.**

Anti-His dot blotting was performed as described in Material and Methods on harvested supernatants. Some additives were found to significantly increase the overall expression of secreted MD proteins (hMD-2, HE82-89 MD-2 chimera, eMD-2 or hMD-1). The effect of additives on protein expression was analysed in a given *Pichia* strain (GS115 or KM71H) and culture medium (BMMY, MM or WMVIII).

**Supplementary Figure 3: Effect of culture media additives on hMD-2 expression**. An hMD-2-expressing GS115 or KM71H colony was induced in the presence of a panel of anti-aggregation agents, and a sample of harvested supernatant was analyzed by dot blotting. The signal intensity from each dot was quantified as a percent of the total signal for each blot by ImageJ®, and this integrated density was plotted for each anti-aggregation agent tested, in both buffered complex medium **(a)** and minimal medium **(b)** for expression in GS115, and in both buffered complex medium **(c)** and minimal medium **(d)** for expression in KM71H.

**c)**

**d)**

**a)**

**b)**

**Supplementary Figure 4: Effect of culture media additives on HE82-89 expression in GS115 and KM71H**. A HE82-89-expressing GS115 or KM71H colony was induced in the presence of a panel of anti-aggregation agents, and a sample of harvested supernatant was analyzed by dot blotting. The signal intensity from each dot was quantified as a percent of the total signal for each blot by ImageJ®, and this integrated density was plotted for each anti-aggregation agent tested, in both buffered complex medium **(a)** and minimal medium **(b)** for expression in GS115, and in both buffered complex medium **(c)** and minimal medium **(d)** for expression in KM71H.

**c)**

**d)**

**a)**

**b)**

**Supplementary Figure 5: Effect of culture media additives on eMD-2 expression in GS115 and KM71H**. An eMD-2-expressing GS115 or KM71H colony was induced in the presence of a panel of anti-aggregation agents, and a sample of harvested supernatant was analyzed by dot blotting. The signal intensity from each dot was quantified as a percent of the total signal for each blot by ImageJ®, and this integrated density was plotted for each anti-aggregation agent tested, in both buffered complex medium **(a)** and minimal medium **(b)** for expression in GS115, and in both buffered complex medium **(c)** and minimal medium **(d)** for expression in KM71H.

**c)**

**d)**

**a)**

**b)**

**Supplementary Figure 6: Effect of culture media additives on hMD-1 expression in GS115 and KM71H**. A hMD-1-expressing GS115 or KM71H colony was induced in the presence of a panel of anti-aggregation agents, and a sample of harvested supernatant was analyzed by dot blotting. The signal intensity from each dot was quantified as a percent of the total signal for each blot by ImageJ®, and this integrated density was plotted for each anti-aggregation agent tested, in both buffered complex medium **(a)** and minimal medium **(b)** for expression in GS115, and in both buffered complex medium **(c)** and minimal medium **(d)** for expression in KM71H.

**c)**

**d)**

**a)**

**b)**

**Supplementary Figures 7-14. Screening of culture additives, step 2: non-reducing Western blot analysis.**

Anti His Western blotting was performed as described in Material and Methods on harvested supernatants under non-reducing conditions to identify additives with anti-aggregation properties. Some additives were found to significantly decrease the formation of intermolecular disulfide bonds for secreted proteins (hMD-2, HE82-89 MD-2 chimera, eMD-2 or hMD-1), in a given *Pichia* strain (GS115 or KM71H) and culture medium (BMMY, MM or WMVIII). BME stands for β-mercaptoethanol.

**Supplementary Figure 7: Expression of hMD-2 in *Pichia* strain KM71H in the presence of anti-aggregation agents**. A recombinant KM71H*Pichia*colony expressing hMD-2 was induced in the presence of various anti-aggregation agents added to either buffered complex induction medium (BMMY) or minimal induction medium (MM). Samples of supernatants harvested from each small-scale induction trial were analyzed by Western blotting in non-reducing conditions.


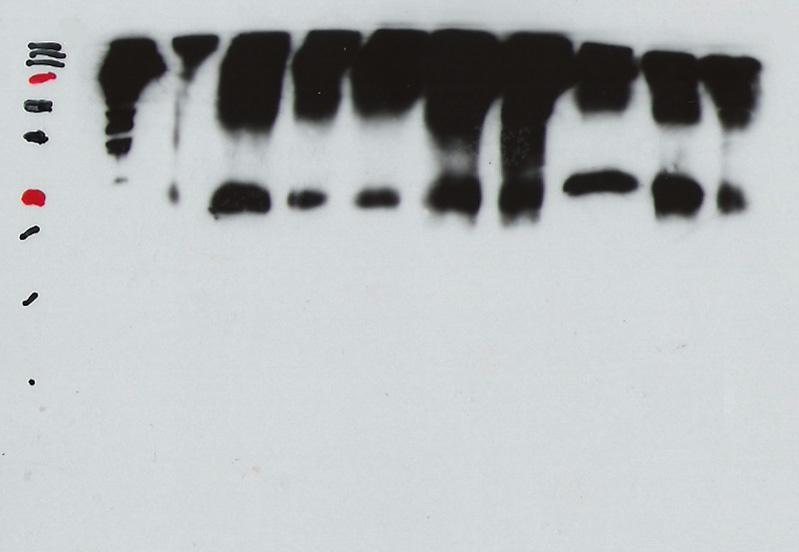

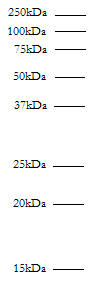


1

2

3

4

5

6

7

8

9

10

**Lane Identifications**

1. MW markers

2. MM

3. 0.1M NaSCN, BMMY

4. 0.5M KCl, BMMY

5. 0.1M CaClR_2_R, BMMY

6. 0.2M MgSOR_4_R, MM

7. 0.1M MgClR_2_R, MM

8. .01mg/mL LPS, BMMY

9. 0.5M NDSB-201, BMMY

10. .01mg/mL LPS, MM


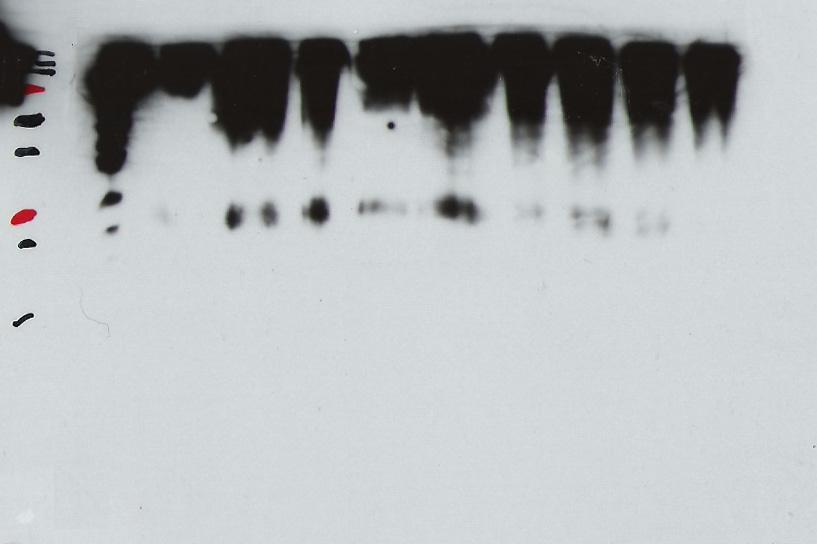

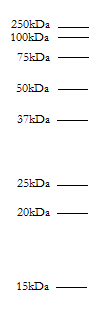


1

2

3

4

5

6

7

8

9

10

**Lane Identifications**

1. MW markers

2. 1% FCS, MM

3. 0.15M (NHR_4_R)R_2_RSOR_4_R, BMMY

4. 0.5M NDSB-201, MM

5. 0.05% Triton X-100, BMMY

6. 1mM CHAPS, MM

7. 5mM OGP, MM

8. 0.05% Pluronic F-68, MM

9. 1% DMSO, MM

10. 1% DMF, MM


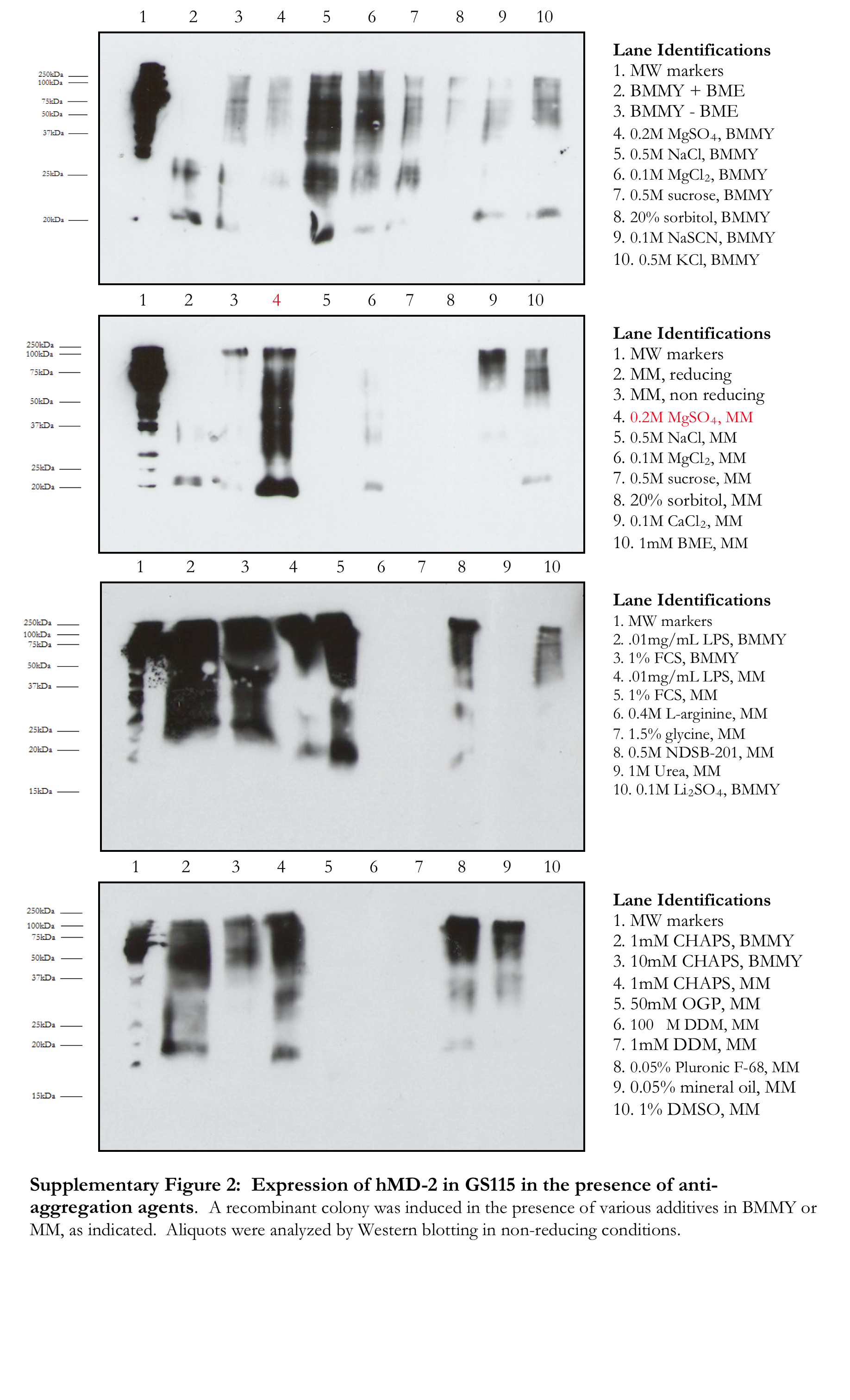


**Supplementary Figure 8: Expression of hMD-2 in GS115 in the presence of anti-aggregation agents**. A recombinant *Pichia* colony expressing hMD-2 was induced in the presence of various anti-aggregation agents added to either buffered complex induction medium (BMMY) or minimal induction medium (MM). Samples of supernatants harvested from each small-scale induction trial were analyzed by Western blotting in non-reducing conditions.

**Supplementary Figure 9: Expression of HE82-89 in *Pichia* strain KM71H in the presence of anti-aggregation agents**. A recombinant KM71H*Pichia* colony expressing HE82-89 was induced in the presence of various anti-aggregation agents added to either buffered complex induction medium (BMMY) or minimal induction medium (MM). Samples of supernatants harvested from each small-scale induction trial were analyzed by Western blotting in non-reducing conditions.


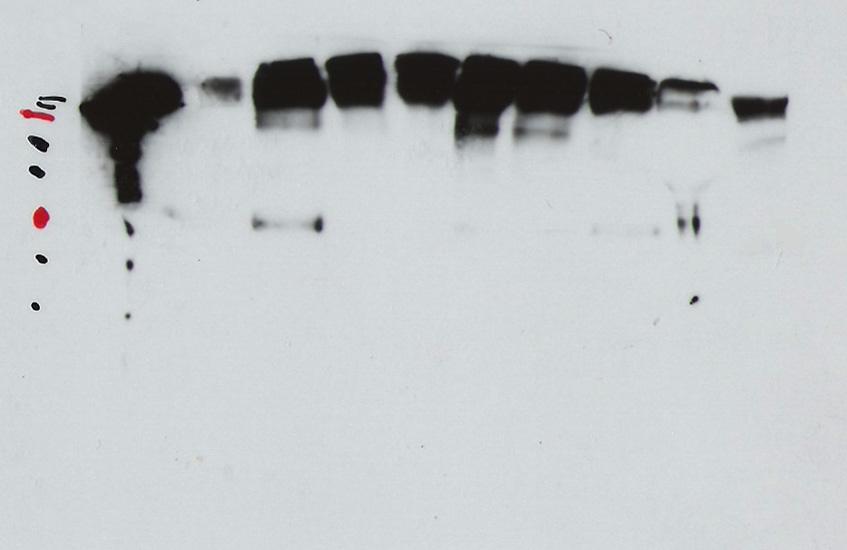

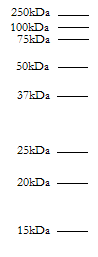


1

2

3

4

5

6

7

8

9

10

**Lane Identifications**

1. MW markers

2. BMMY

3. BMMY, pH 5.5

4. 0.1M CaClR_2_R, BMMY

5. 0.2M MgSOR_4_R, BMMY

6. 0.5M sucrose, BMMY

7. WM8, BMMY

8. .01mg/mL LPS, BMMY

9. 10% FCS, BMMY

10. 0.4M L-arginine, BMMY


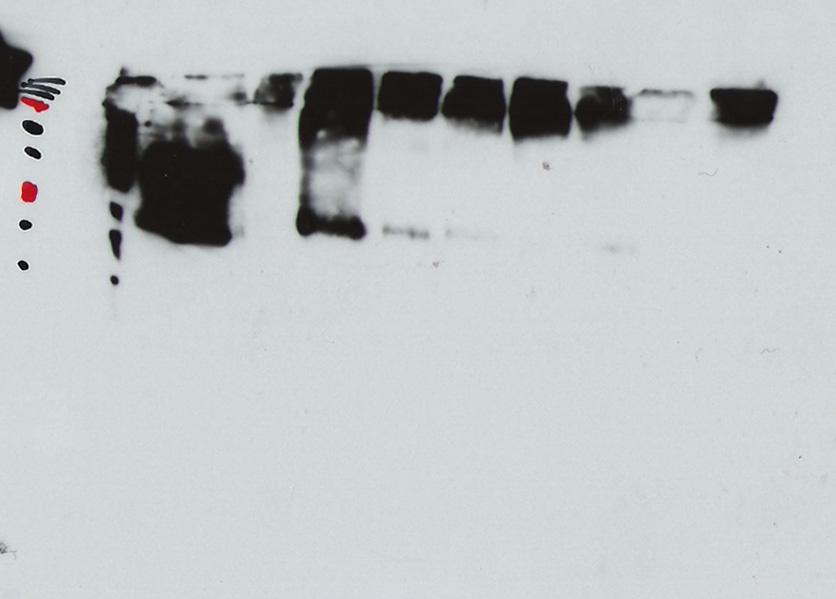

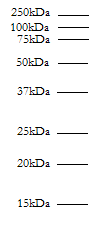


1

2

3

4

5

6

7

8

9

10

**Lane Identifications**

1. MW markers

2. 1.5% glycine, BMMY

3. 0.5M NDSB-201

4. 0.15M(NHR_4_R)R_2_RSOR_4_R, BMMY

5. 0.1M LiR_2_RSOR_4_R, BMMY

6. 1mM CHAPS, BMMY

7. 1mM DDM, BMMY

8. 1% L-glutathione, BMMY

9. 0.05% Pluronic F-68, BMMY

10. 1% DMF, BMMY

**Supplementary Figure 10: Expression of HE82-89 in *Pichia* strain GS115 in the presence of anti-aggregation agents**. A recombinant GS115 *Pichia* colony expressing HE82-89 was induced in the presence of various anti-aggregation agents added to either buffered complex induction medium (BMMY) or minimal induction medium (MM). Samples of supernatants harvested from each small-scale induction trial were analyzed by Western blotting in non-reducing conditions.


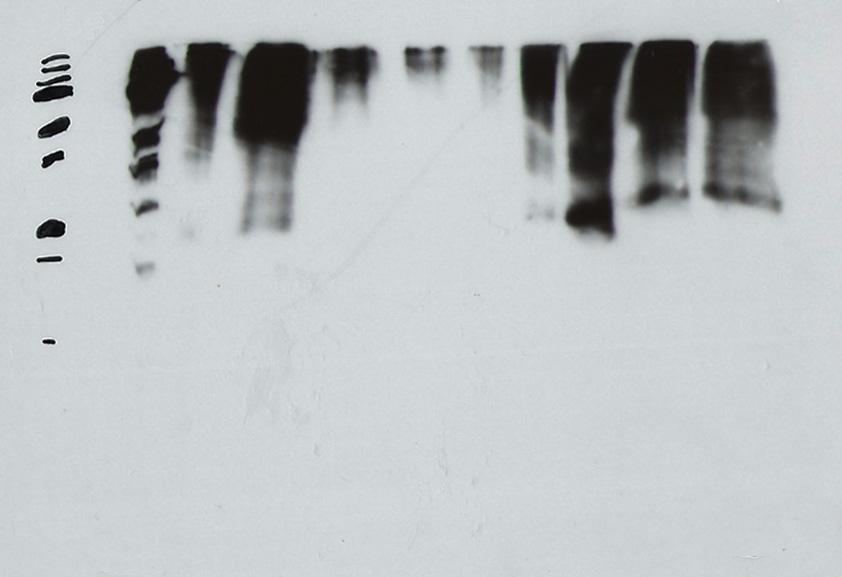

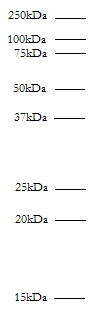


1

2

3

4

5

6

7

8

9

10

**Lane Identifications**

1. MW markers

2. MM

3. 0.1M NaSCN, BMMY

4. 0.2M MgSOR_4_R, MM

5. 0.1M MgClR_2_R, MM

6. 0.5M sucrose, MM

7. 20% sorbitol, MM

8. WM8, MM

9. 0.5M NDSB-201, BMMY

10. 1.5% glycine, BMMY


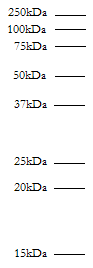

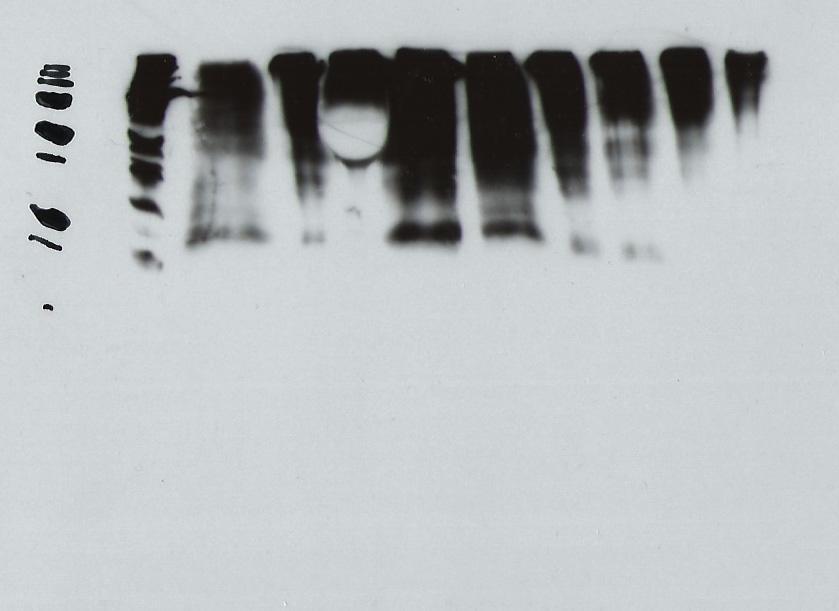


1

2

3

4

5

6

7

8

9

10

**Lane Identifications**

1. MW markers

2. 0.4M L-arginine, BMMY

3. .01mg/mL LPS, MM

4. 10% FCS, MM

5. 0.15M (NHR_4_R)R_2_RSOR_4_R, BMMY

6. 1mM DDM, BMMY

7. 1mM CHAPS, MM

8. 0.05% Triton X-100, MM

9. 0.05% Pluronic F-68, MM

10. 0.05% Mineral oil, MM

**Supplementary Figure 11: Expression of eMD-2 in *Pichia* strain KM71H in the presence of anti-aggregation agents**. A recombinant KM71H*Pichia* colony expressing eMD-2 was induced in the presence of various anti-aggregation agents added to either buffered complex induction medium (BMMY) or minimal induction medium (MM). Samples of supernatants harvested from each small-scale induction trial were analyzed by Western blotting in non-reducing conditions.


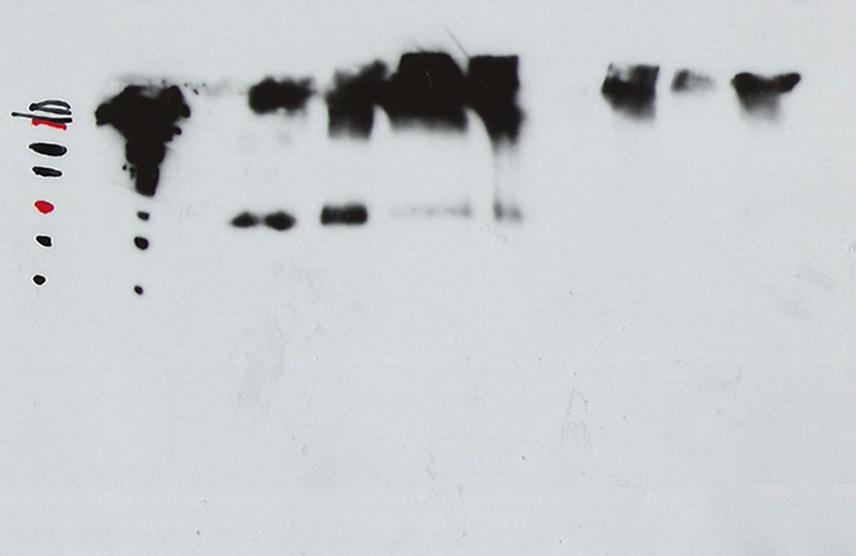

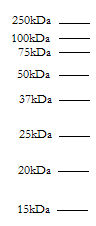


1

2

3

4

5

6

7

8

9

10

**Lane Identifications**

1. MW markers

2. MM

3. 0.1M NaSCN, BMMY

4. 0.5M KCl, BMMY

5. 0.1M CaClR_2_R, BMMY

6. 0.2M MgSOR_4_R, MM

7. 0.5M NaCl, MM

8. 0.1M MgClR_2_R, MM

9. 20% sorbitol, MM

10. .01mg/mL LPS, MM


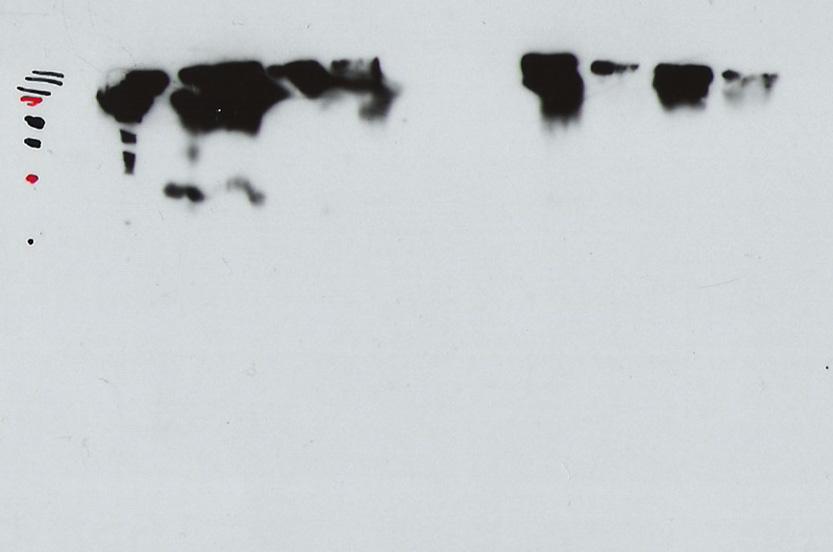

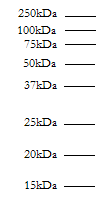


1

2

3

4

5

6

7

8

9

10

**Lane Identifications**

1. MW markers

2. 0.15M (NHR_4_R)R_2_RSOR_4_R, MM

3. 1% FCS, MM

4. 0.5M NDSB-201, MM

5. 1mM DDM, MM

6. 1% L-glutathione, MM

7. 0.05% Pluronic F-68, MM

8. 10mM CHAPS, MM

9. 0.05% mineral oil, MM

10. 1% DMF, MM


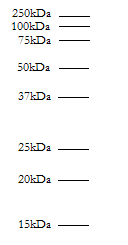

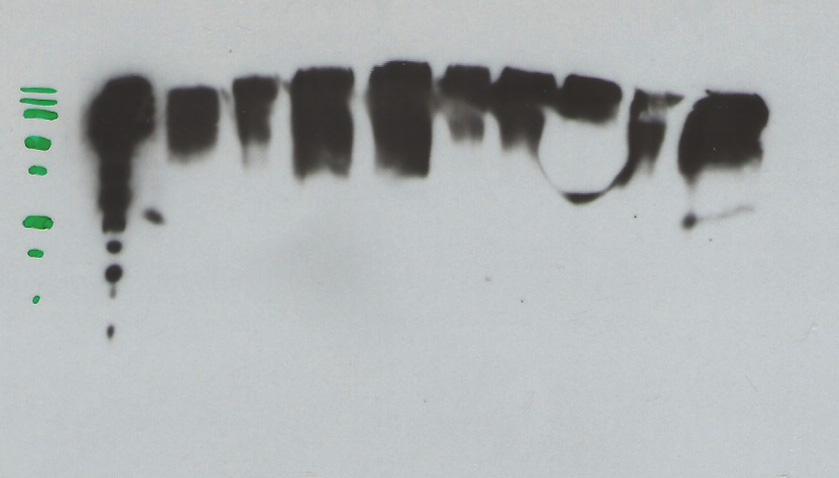


1

2

3

4

5

6

7

8

9

10


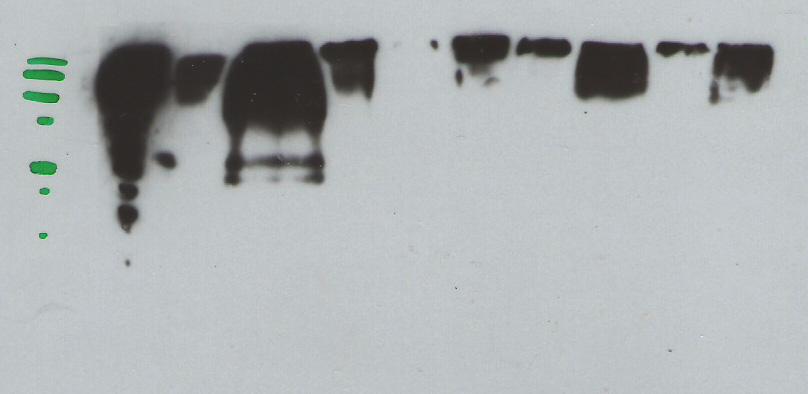

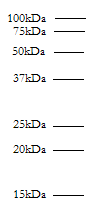


1

2

3

4

5

6

7

8

9

10

**Supplementary Figure 12: Expression of eMD-2 in *Pichia* strain GS115 in the presence of anti-aggregation agents**. A recombinant GS115 *Pichia* colony expressing eMD-2 was induced in the presence of various anti-aggregation agents added to either buffered complex induction medium (BMMY) or minimal induction medium (MM). Samples of supernatants harvested from each small-scale induction trial were analyzed by Western blotting in non-reducing conditions.

**Lane Identifications**

1. MW markers

2. BMMY

3. MM

4. 0.2M MgSOR_4_R, MM

5. 0.1M MgClR_2_R, MM

6. 20% sorbitol, MM

7. .01mg/mL LPS, MM

8. 10% FCS, MM

9. 0.15M (NHR_4_R)R_2_RSOR_4_R, MM

10. .01mg/mL LPS, BMMY

**Lane Identifications**

1. MW markers

2. 1.5% glycine, BMMY

3. 0.5M NDSB-201, BMMY

4. 1mM CHAPS, MM

5. 10mM CHAPS, MM

6. 1% L-glutathione, MM

7. 0.05% Pluronic F-68, MM

8. 0.05% Pluronic F-68, BMMY

9. 1% DMF, MM

10. 1% DMSO, BMMY

**Supplementary Figure 13: Expression of hMD-1 in *Pichia* strain KM71H in the presence of anti-aggregation agents**. A recombinant KM71H *Pichia* colony expressing hMD-1 was induced in the presence of various anti-aggregation agents added to either buffered complex induction medium (BMMY) or minimal induction medium (MM). Samples of supernatants harvested from each small-scale induction trial were analyzed by SDS-PAGE followed by Western blotting in non-reducing conditions.

**Lane Identifications**

1. MW markers

2. MM

3. 0.1M CaClR_2_R, BMMY

4. 0.1M MgClR_2_R, BMMY

5. 0.2M MgSOR_4_R, MM

6. 0.5 NDSB-201, BMMY

7. .01mg/mL LPS, BMMY

8. 20% sorbitol, MM

9. 0.1M MgClR_2_R, MM

10. .01mg/mL LPS, MM

**Lane Identifications**

1. MW markers

2. 10% FCS, MM

3. 0.5M NDSB-201, MM

4. 0.15M (NHR_4_R)R_2_RSOR_4_R, MM

5. 1mM CHAPS, MM

6. 100 μM DDM, MM

7. 1% glutathione, MM

8. 0.05% Pluronic F-68, MM

9. 1% DMSO, MM

10. 100 μM DDM, BMMY


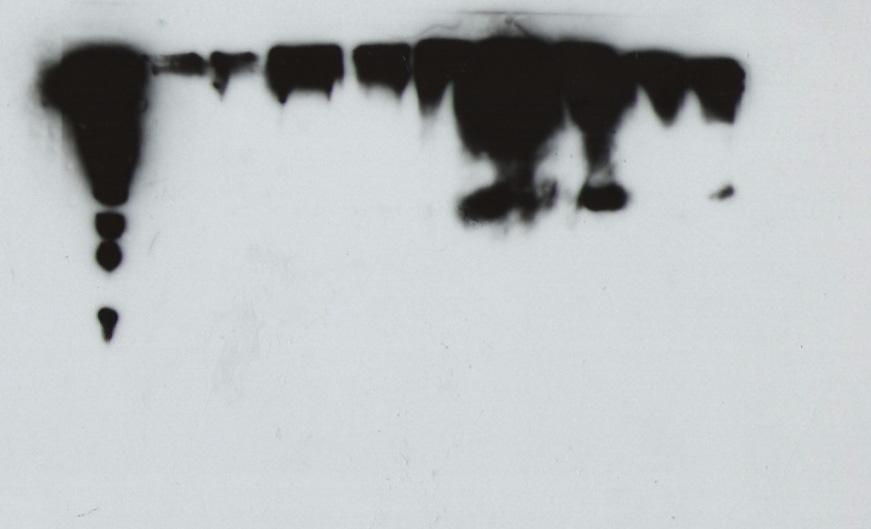

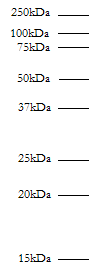


1

2

3

4

5

6

7

8

9

10


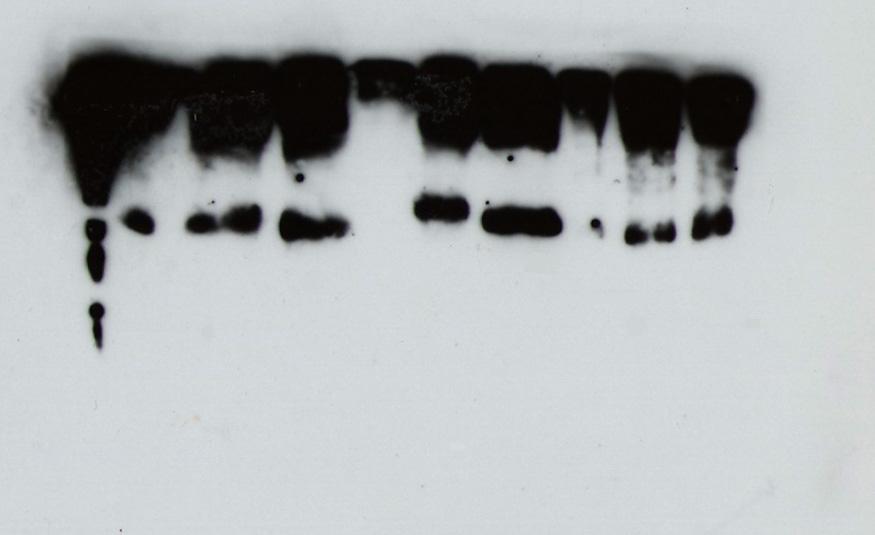

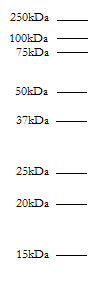


1

2

3

4

5

6

7

8

9

10

**Supplementary Figure 14: Expression of hMD-1 in *Pichia* strain GS115 in the presence of anti-aggregation agents**. A recombinant GS115 *Pichia* colony expressing hMD-1 was induced in the presence of various anti-aggregation agents added to either buffered complex induction medium (BMMY) or minimal induction medium (MM). Samples of supernatants harvested from each small-scale induction trial were analyzed by SDS-PAGE followed by Western blotting in non-reducing conditions.


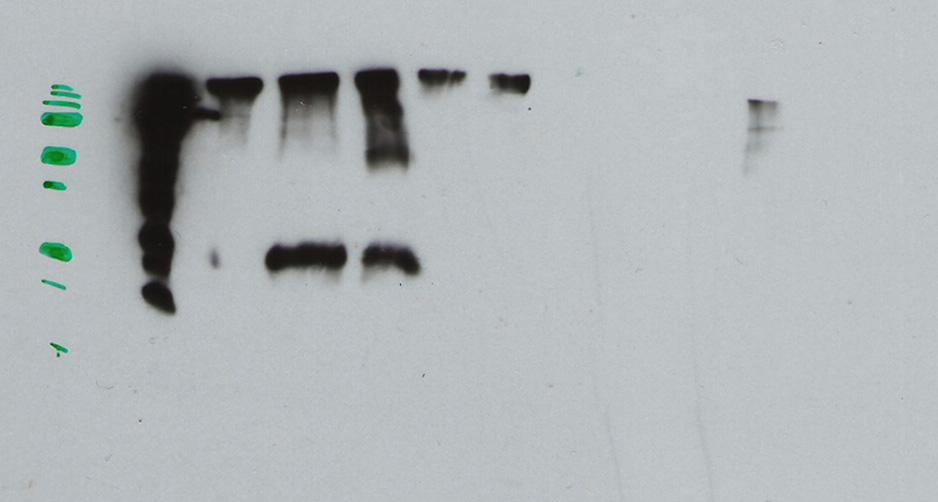

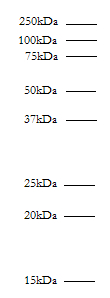


1

2

3

4

5

6

7

8

9

10


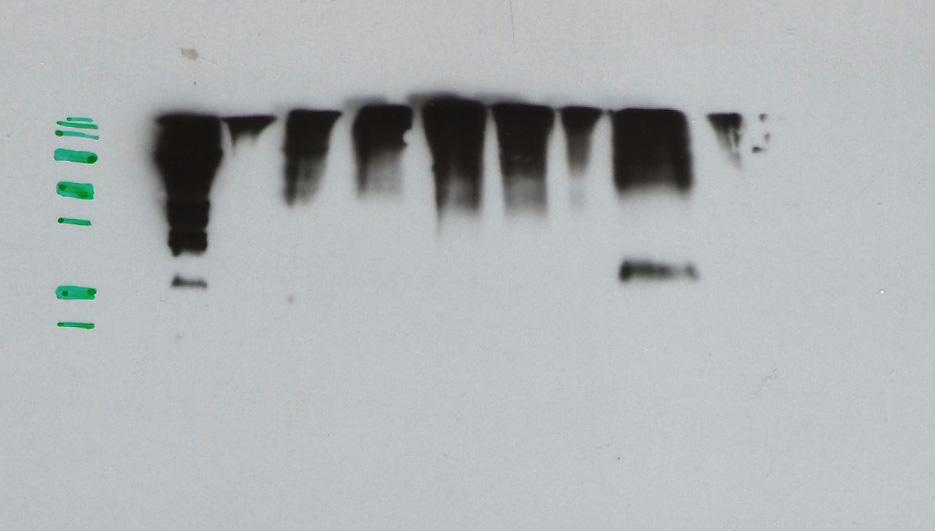

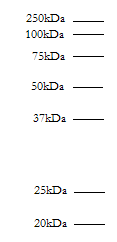


1

2

3

4

5

6

7

8

9

10

**Lane Identifications**

1. MW markers

2. 0.15M (NHR_4_R)R_2_RSOR_4_R, MM

3. 1.5% glycine, BMMY

4. 0.5M NDSB-201, BMMY

5. 0.05% TritonX-100, MM

6. 1mM CHAPS, MM

7. 0.05% Pluronic F-68, MM

8. 1% DMF, MM

9. 1% L-glutathione, MM

10. 1% DMSO, BMMY

**Lane Identifications**

1. MW markers

2. MM

3. BMMY, pH 5.5

4. 0.1M CaClR_2_R, BMMY

5. 0.2M MgSOR_4_R, MM

6. 0.1M MgClR_2_R, MM

7. 20% sorbitol, MM

8. .01mg/mL LPS, BMMY

9. .01mg/mL LPS, MM

10. 10% FCS, MM
